# Endoplasmic reticulum-mitochondria tethering in neurodegenerative diseases

**DOI:** 10.1186/s40035-017-0092-6

**Published:** 2017-08-23

**Authors:** Yi Liu, Xiongwei Zhu

**Affiliations:** Department of Pathology, Case Western Reserve University, Cleveland, OH USA

**Keywords:** Mitochondria-associated ER membrane, Mitochondria-ER tethering, Alzheimer’s disease, Parkinson’s disease, Amyotrophic lateral sclerosis

## Abstract

Endoplasmic reticulum (ER) and mitochondria are tubular organelles with a characteristic “network structure” that facilitates the formation of inter-organellar connections. As a result, mitochondria-associated ER membranes (MAMs), a subdomain of the ER that is tightly linked to and communicates with mitochondria, serve multiple physiological functions including lipid synthesis and exchange, calcium signaling, bioenergetics, and apoptosis. Importantly, emerging evidence suggests that the abnormality and dysfunction of MAMs have been involved in various neurodegenerative disorders including Alzheimer’s disease, amyotrophic lateral sclerosis, and Parkinson’s disease. This review will focus on the architecture and function of MAMs and its involvement in the neurodegenerative diseases.

## Background

In 1990, Vance [1] found that crude rat liver mitochondrial fraction was capable of rapid and linked synthesis of phospholipids and also contained phosphatidylserine synthase (PSS) and phosphatidylethanolamine methyltransferase activities which were absent from highly purified mitochondria. This could be attributed to the presence of a membrane fraction ‘X’ in the crude but not highly purified mitochondria. It turns out that this fraction ‘X’ , now commonly regarded as the mitochondrial associated membrane (MAMs), is a specialized subdomain of the endoplasmic reticulum (ER) with specific lipid and protein composition that is involved in the crosstalk with mitochondria. Phospholipid synthesis and exchange was the first identified function of MAMs. Later, it was demonstrated by electron tomography that ER and mitochondria are linked by high electron-dense tethers (10 nm at the smooth ER and 25 nm at the rough ER) termed as mitochondria-ER tethers (a.k.a., *M*itochondria-*ER* *c*ontact/*c*rosslink/*c*rosstalk/*c*ommunication or MERC) [2, 3]. In fact, approximately 5 ~ 20% of the mitochondrial surface is juxtaposed (10–30 nm distances) to specialized regions of the ER tubules [4–7]. Such a short distance suggests that the two organelles are tethered together by proteins on the juxtaposed membranes [7]. MERC appear to be stable structures. Although the ER and mitochondrial membranes form multiple and specific crosslink sites, it must be emphasized that they do not fuse but instead still maintain the organelles’ distinct structures and characteristics which is crucial for them to serve as a major platform to carry out important physiological roles in the regulation of intracellular calcium homeostasis [6, 8–12], lipid metabolism [1, 13], mitochondrial fission [14], autophagosome formation [15] and apoptosis progression [16, 17]. In this review, we will focus on recent data addressing the structural composition and function of the MAMs in mammalian system and its potential involvement in various neurodegenerative diseases.

## ER–mitochondria tethering protein complexes

**Fig. 1:**
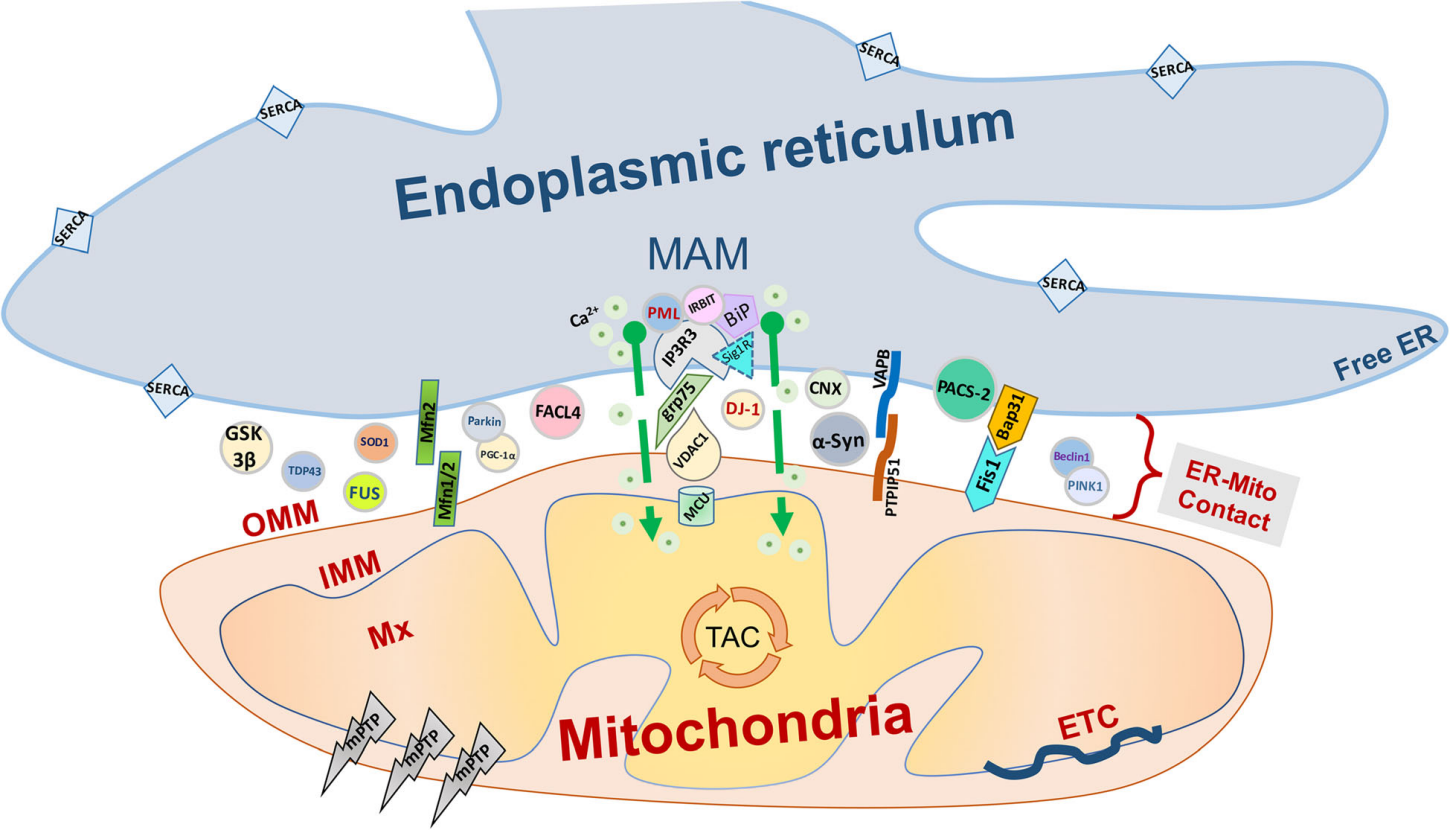
Global view of the architecture/choreography of ER–mitochondria contacts. As depicted, a part of ER tubule and mitochondria form quasi-synaptic structure. Several pairs of integral membrane proteins located on mitochondria and ER important for MERC formation and physical tethering of the organelles were identified, including Mfn1/2 tether, Fis1-Bap31 tether, VAPB-PTPIP51 tether and IP3R-grp75-VDAC1 tripartite complex. The latter is essential for the efficient Ca^2+^ transfer from the ER to mitochondria. MAM: mitochondria associated ER membrane, OMM = outer mitochondrial membrane, IMM: inner mitochondrial membrane, Mx = matrix, ETC: electron transport chain, TAC: tricarboxylic acid cycle

The molecular details of ER–mitochondria tethers are largely unknown. Electron microscopic studies showed that the cleft of a MERC is typically dotted by electron-dense areas that are widely accepted to be formed by protein complexes. The MAM fraction can be detached from mitochondria through the limited digestion with trypsin or Proteinase K which is suggestive of proteinaceous characteristics [7]. Indeed, several pairs of integral membrane proteins located on mitochondria and ER important for MERC formation and physical tethering of the organelles were identified (Fig. 1) [5]. Additional proteins that localize in the cleft of the MERC and participate in its biochemical activity or functional regulation might also, directly or indirectly, participate in MERC formation or maintenance such as by spacing apart the opposing membranes of the ER and the mitochondrion at a distance that allows the formation of MERC tethers.

1. **Mfn2 Tether**
  Mitofusin 2 (Mfn2) is a large GTPase involved in the fusion of mitochondrial outer membrane. After the initial report from Luca Scorrano’s group suggesting the potential involvement of Mfn2 in ER-mitochondrial tethering, Mfn2 is among the most widely studied ER-mitochondria tethering protein factors but its exact role in MERC regulation is still in hot debate. In 2008, Luca Scoranno and colleagues found that Mfn2 ablation caused dramatic defects in ER morphology in vitro in mouse embryonic fibroblasts(MEFs) and HeLa cells [18]. It turned out that Mfn2 is enriched at MERC and is required for the juxtaposition of ER to mitochondria [18]. ER-located Mfn2 interacts *in trans* with mitochondrial mitofusins (i.e., Mfn1 or Mfn2) to form trans-organelle hetero- or homo- dimer tethers to bridge the mitochondria onto ER which allows efficient calcium transfer between ER and mitochondria [18, 19]. The confocal microscopy analysis revealed that the distance between ER and mitochondria increases in cells lacking Mfn2 which impairs mitochondrial calcium uptake [18, 19]. Indeed, the presence and enrichment of Mfn2 in the interface between ER and mitochondria is directly demonstrated by immunoelectron microscopy in Melanocytes [20]. Moreover, Mfn2-mediated ER-mitochondria tethering is regulated by MitoL-dependent activation of mitochondrial Mfn2 through ubiquitination and MitoL ablation inhibited Mfn2 complex formation and caused Mfn2 mislocalization from MAM to non-MAM ER [21]. Owing to its well-known role in the tethering of adjacent membranes, Mfn2 is widely accepted as a major regulator of MERC in different tissues [18, 19, 22]. However, such a view was challenged by a quantitative electron microscopic study followed by a series morphological and functional studies from multiple groups: In 2012, Pierre C et al. reported that the percentage of mitochondria in close contact with ER tubules was increased in Mfn2 defective MEFs when compared to wildtype MEFs (4.91% vs 2.25%, the distance between ER and mitochondria was restricted to <20 nm in the study). In 2015, Riccardo Filadia et al. confirmed this EM findings that the ER-mitochondria crosslink was increased in Mfn2 defective MEFs and SH-SY5Y cells and provided functional evidence of an elevated calcium flow from ER to mitochondria in Mfn2 defective MEFs [23]. While they also confirmed the confocal microscopy results from Scoranno’s group that Mfn2 deficiency caused a net decrease in the overlapping area between ER and mitochondria, they noted that the dramatic changes in mitochondrial morphology caused by Mfn2 ablation resulted in changes in mitochondrial area which could make classical colocalization analysis unsuitable. Excluding the confounding effects of area change, they found that mitochondrial perimeter colocalizing with the ER actually increased in Mfn2 deficient cells [23]. Other groups also reported that Mfn2 knockdown increased ER-mitochondria tethering and calcium transfer from ER to mitochondria [24]. Along this line, deficiency in Gp78 (a.k.a. autocrine motility factor receptor, AMFR), an endoplasmic-reticulum (ER)-associated protein degradation E3 ubiquitin ligase involved in the degradation of Mfn2, caused significantly decreased rough ER-mitochondria crosstalk as evaluated by EM which could be blocked by Mfn2 knockdown, suggesting that Gp78 might promote ER-mitochondria interaction through degradation of Mfn2 [25]. Collectively, these studies strongly challenges the role of Mfn2 as an essential component of ER-mitochondrial tethering but argues for a role of Mfn2 as a negative regulator of organelle apposition. Most recently, Luca Scorrano’s group responded to these challenges by providing further ultramorphometric and confocal microscopic evidence based on unbiased fluorescent probes of ER-mitochondrial proximity to demonstrate that Mfn2 ablation increases ER-mitochondria distance which resulted in impaired mitochondrial calcium uptake in an mitochondrial calcium uniporter (MCU)-independent manner in MEF cells [22], yet the number of ER-mitochondria contacts were not directly addressed in this study and it is not clear whether and how larger ER-mitochondrial distance may affect ER-mitochondrial tethering. Further research is needed to fully elucidate the role of Mfn2 at MERC.
2. **VAPB-PTPIP51 Tether**
  Vesicle-associated membrane protein-associated protein B (VAPB) is an integral protein in the ER membrane involved in ER unfolded protein response and the regulation of cellular calcium homeostasis [26]. Most recently, it was demonstrated that VAPB interacts with mitochondrial outer membrane protein tyrosine phosphatase-interacting protein-51 (PTPIP51) [27]. Overexpression of either proteins increase, while knockdown of either protein decreases, ER-mitochondria tethering along with functional changes such as calcium exchange between the two organelles, suggesting that this pair of proteins forms another molecular scaffold to tether the two organelles [28].
3. **Fis1-Bap31 Tether**
  As an ER protein-sorting factor, B-cell receptor-associated protein 31 (Bap31), an abundant 28-kDa integral membrane chaperone protein of the ER, forms several large protein complexes and controls the fate of newly synthesized integral membrane proteins. An earlier study demonstrated that outer membrane associated active caspase 8 could cleave ER-associated Bap31 and the cleavage product p20Bap31 mediated mitochondrion-ER cross talk through a calcium-dependent mechanism [29]. The overexpression of P20Bap31 could lead to early release of calcium from ER and concomitant uptake of calcium into mitochondria [30], implicating the functional involvement of Bap31 in ER-mitochondrial tethering. More recently, it was demonstrated that mitochondrial fission protein Fission 1 homologue (Fis1) interacts with Bap31 and forms an ER-mitochondrial platform which is essential in the recruitment and activation of procaspase 8 and the conveyance of the apoptotic signal from mitochondria to ER [31]. Given that Fis1-Bap31 interaction is also present in normal, non-apoptotic cells [31], it likely constitutes a preformed scaffold complex to tether ER-mitochondria together that may have roles broader than apoptotic signaling. The role of Bap31 in ER-mitochondria tethering may be modulated by phosphofurin acidic cluster sorting protein 2 (PACS-2), a multifunctional cytosolic sorting protein: depletion of PACS-2 caused Bap-31-dependent mitochondrial fragmentation and uncoupling from the ER along with inhibition of calcium signal transmission [32]. More recently, it was demonstrated that mammalian target of rapamycin complex 2 regulates the integrity of MAM by Akt-dependent phosphorylation of PACS-2 [33] although how PACS-2 promotes MAM integrity and regulates its composition is not entirely clear.
4. **IP3R3-Grp75-VDAC1**
  The voltage-dependent anion channel (VDAC) of the outer mitochondrial membrane interacts with the ER calcium-release channel inositol 1,4,5-trisphosphate receptor (IP3R) through the molecular chaperone glucose-regulated protein 75 (grp75), which is essential for the efficient calcium transfer from the ER to mitochondria [34, 35]. Despite such an important functional role, this tripartite complex may not have a tethering role, but rather a MERC spacing/filling function that derives from functionally coupling ER and mitochondria in calcium exchanges. Nevertheless, the ER-resident protein Sigma1R (Sig-1R) stabilizes MAM by interacting with VDAC and IP3R and prolongs the calcium signaling in MAMs [36].

## Functions of MAM

Mitochondria and ER have crucial but distinctive roles in mammalian cells. ER-mitochondria tethering provides a platform facilitating the crosstalk between these two organelles which appears essential for the function of these organelles. Indeed, it becomes increasingly clear that MAM plays an important role in various cellular processes critical for cell survival and death, which is briefly discussed below.

1. **Phospholipid Synthesis and Exchange**
  While lipid synthesis largely occurs in the ER, it needs the assistance of other organelles since several of the key enzymes are located on the membrane of other organelles such as mitochondria [13, 37]. MAMs were initially described as an ER subdomain enriched in proteins involved in lipid metabolism such as PSS1 and PSS2 with an ascribed function as a platform for lipid biosynthesis and exchange [1, 38]. Indeed, phosphatidylserine is first synthesized in the MAM by PSS1 and PSS2 [39]; then transferred to the closely apposed mitochondrion, where a decarboxylase in the inner mitochondrial membrane converts it to phosphatidylethanolamine; to make phosphotidylcholine, phosphatidylethanolamine is transferred back to the ER, where a methyltransferase converts it into phosphatidylcholine, a major component of the cell membrane. Phosphotidylcholine must be transferred back to mitochondria again since it is present in mitochondrial membrane [13]. Later studies revealed that MAMs are also the site of triacylglycerol synthesis and steroidogenesis [40]. Another lipid metabolism associated enzyme enriched in MAMs is Long-chain-fatty-acid-CoA ligase 4 (FACL4), also known as acyl-CoA synthetase long-chain family member 4, which mediates the ligation of fatty acids to coenzyme A (CoA) and other cholesterol metabolites. Acyl-coenzyme A:cholesterol acyltransferase-1 that catalyzes the formation of cholesterol esters and diacylglycerol acyltransferase are also enriched in MAMs [41, 42].
2. **Calcium Transfer**
  The calcium homeostasis of mitochondria is of vital significance to mitochondria and cell as a whole. On one hand, moderate loading of mitochondrial calcium has an important physiological function to stimulate ATP production through calcium-dependent activation of the key metabolic enzymes in the Krebs cycle such as pyruvate dehydrogenase and α-ketoglutarate dehydrogenase [43]. On the other hand, calcium overloading of mitochondria causes the opening of mitochondrial permeability transition pore (mPTP) and leads to cell apoptosis [44]. ER is the intracellular calcium store and MAMs facilitate the calcium transfer between ER and mitochondria [9, 11]. While outer mitochondrial membrane is permeable to calcium through VDAC1, the inner mitochondrial membrane is not and calcium needs to go through MCU in the inner mitochondrial membrane [45–47]. As a low affinity calcium channel, MCU requires high calcium levels which can be efficiently achieved by the MAM through IP3R3-grp75-VDAC1 complex: the calcium released from ER through IP3R to the VDAC1 on the OMM leads to localized sites of calcium influx in the intermembrane space and creates the microdomain of high [Ca^2+^] (calcium puff) close to MCU and thus facilitate the calcium uptake by MCU [48, 49]. The IP3R3-grp75-VDAC1 complex also serves as a molecular scaffold for other calcium handling players (Sig-1R [36], BiP, Bcl-2 [50], IRBIT [51], etc.), which may be essential for the fine-tuning of the calcium signaling of IP3R3-grp75-VDAC1 axis at the MAM.
3. **Regulation of Mitochondrial Fission**
  Mitochondria are highly dynamic organelles that undergo continuous fusion and division which is critical for the homeostasis of mitochondria [52]. Mitochondrial fission involves the translocation of cytosolic dynamin-like protein (DLP1) to the outer membrane of mitochondria where it oligomerizes into ring-like structures that wrap around the mitochondrial constriction sites [14, 53, 54]. Interestingly, during fission, mitochondria appeared to be constricted at the point of contact with the ER. In both yeast and mammalian cells, Dnm1 (DLP1 yeast ortholog)/DLP1 localizes to the mitochondrial membrane constriction site wrapped by ER tubules [14]. Three-dimensional electron microscopic study of the structure of MERC in *Saccharomyces cerevisiae* revealed that mitochondria-associated ER tubules might mediate the formation of mitochondrial constriction sites [14]. The presence of ER tubules at mitochondrial constriction and fission sites has been further evidenced using a two-color STORM super-resolution study [55]. In fact, the perimeter of mitochondria tubules circumscribed by ER decreased by ~30% when compared with the uncircumscribed which better fit for the action of narrower DLP1 ring [14]. A later study revealed that the ER-localized protein inverted formin 2 is activated during fission to polymerize actin, which in turn might generate the driving force for initial mitochondrial constriction at the ER-mitochondria contact site [56].
4. **Apoptosis**
  Both mitochondria and ER play a role in apoptosis and MERC provides a platform for the two organelles to exchange signals to regulate and coordinate a rapid and orderly cell demise [57]. The Fis1-Bap31 tethering complex (as discussed in “Fis1-Bap31 tether” earlier) allows for apoptotic signals being transferred from mitochondria to the ER by recruiting and activating caspase 8 which cleaves Bap31 into the pro-apoptotic p20Bap31 [31]. P20Bap31 propagates the apoptotic signal from ER back to mitochondria by stimulating the ER calcium release which activates and opens the mPTP and thus amplifies the death signal [58]. IP3R-Grp75-VDAC1 complex at the MAM is crucial for mitochondrial calcium uptake which could thus participate in the apoptotic signal through mitochondrial calcium overload. SUMOylation of DLP1 was also reported to stabilize the MERC and promote calcium crosstalk and cytochrome c release [59].

## Disturbed ER-mitochondria tethering in neurodegenerative disorders

Alzheimer’s disease (AD), Parkinson’s disease (PD), and amyotrophic lateral sclerosis with associated frontotemporal dementia (ALS/FTD) are major neurodegenerative diseases. These neurodegenerative diseases are characterized by damage to various cellular processes, many of which are regulated by ER-mitochondria tethering. Indeed, abnormal MAM signaling is reported in all these neurodegenerative diseases as reviewed below.

1. **Alzheimer’s Disease**
  Presenilins are the catalytic subunits of the gamma secretase responsible for the generation of amyloid-β (Aβ) [60]. Mutations in presenilin 1 (PS1) and 2 (PS2) cause human familial AD. Presenilins were present in many compartments in the cell including ER and mitochondria [61]. Eric Schon’s group first demonstrated the enrichment of presenilins in the MAM and suggested that MAM is the predominant subcellular location for PS1/PS2 and gamma secretase activity and later further demonstrated significantly increased MAM function and ER-mitochondrial communication in presenilin-deficient cells and in fibroblasts from patients with both the familial and sporadic forms of AD, suggesting that upregulated MAM function and increased ER-mitochondria crosstalk may be involved in the pathogenesis of AD [60, 62, 63]. Indeed, expression of MAM-associated proteins were significantly increased in postmortem brain of human AD patients and in the APP transgenic mice although direct evidence of specific alterations in the ER-mitochondria interaction was lacking [64]. Consistent with this notion, Aβ treatment leads to increased MAM protein expression and increased MERC in cultured neurons and ApoE4 allele also upregulated MAM function [65]. However, another group, although agreeing upon the notion that presenilin modulates MAM function, disagreed on the specific effects of PS2 on MAM: Pizzo’s group reported that the expression of the PS2, but not of PS1, facilitates the physical interaction and functional coupling between ER and mitochondria in Mfn2-dependent manner [66]. The reason for the discrepancy is not clear.
2. **Amyotrophic Lateral Sclerosis with associated Frontotemporal Dementia**
  Mutations in genes encoding several different MAM proteins, including a tethering protein, were associated with familial ALS which provides strong evidence to support the involvement of disturbed MAM function in the pathogenesis of ALS although it is hard to reconcile whether upregulated or disrupted MAM function plays the pathogenic role. VAPB interacts with the OMM protein PTPIP51 to form a MAM tether complex [27]. P56S mutation of VAPB causes autosomal-dominant ALS and VAPB-P56S mutant demonstrated higher affinity for PTPIP51 and consequently, increased Ca^2+^ transfer into mitochondria [27]. On the other hand, Sig-1R is another MAM protein that participates in the IP3R function to facilitate calcium signaling at MAM [36]. Mutations in Sig-1R caused juvenile ALS although in an autosomal recessive pattern [67]. Loss of Sig-1R function by gene mutation or downregulation has been shown to break ER–mitochondria associations [68]. ALS-causing superoxide dismutase 1 (SOD1) mutants aggregated and accumulated at MAM which compromised the integrity and activity of MAM [69]. ALS/FTD-associated TAR DNA-binding protein 43 (TDP-43), and fused in sarcoma (FUS) could also disrupt the MERC and perturb mitochondrial calcium uptake from ER through activation of glycogen synthase kinase 3β (GSK3β) which in turn perturbs VAPB-PTPIP51 interaction [28].
3. **Parkinson’s Disease**
  Mutations in α-synuclein gene cause autosomal dominant PD and α-synuclein aggregates were major components of Lewy bodies. α-synuclein is present in MAM [70, 71], but pathogenic point mutations of α-synuclein result in its reduced association with MAM, coincident with a lower degree of apposition of ER with mitochondria, a decrease in MAM function, and an increase in mitochondrial fragmentation. Mutations in PARK2 gene (encoding Parkin protein) is associated with juvenile onset autosomal recessive forms of PD. Parkin is ATF4-dependently upregulated during mitochondrial and ER stress, which regulates the functional interplay between ER and mitochondria to help promote cell survival under stress [72]. Interestingly, Parkin is translocated to endoplasmic reticulum (ER) and mitochondrial/ER junctions following excitotoxicity, implicating a potential role for Parkin in MAM [73]. Indeed, a confocal microscopic study showed that Parkin overexpression resulted in enhanced physical coupling between ER and mitochondria and favored calcium transfer from the ER to the mitochondria following 1,4,5 inositol trisphosphate (InsP3) generating agonist and increased the agonist-induced ATP production in vitro [74]. Such a positive effect of Parkin on the ER-mitochondria interaction has been confirmed in nigral neurons by EM analysis [75]. However, a recent study demonstrated enhanced ER-mitochondria tethering in primary fibroblasts from Parkin knockout mice and PD patients with PARK2 gene mutations as well as in neurons derived from induced pluripotent stem cells of a patient with PARK2 gene mutations along with ER-to-mitochondria calcium transfers likely due to increased Mfn2 in MAMs [76]. PINK1, another familial PD and mitochondria quality control associated protein, and the pro-autophagic protein BECN1/Beclin1 were both found at MAM, the PINK1 and Beclin1 interaction enhanced ER-mitochondria contact and promoted the formation of autophagosome following mitophagy induction [77]. Mutations or deletion of DJ-1 are associated with autosomal recessive early onset familial PD. It was reported that DJ-1 is also present in the MAMs. DJ-1 overexpression caused increased Mitochondria-ER colocalization as demonstrated by confocal microscopy [78]. Whether this represents true increase in the MERC needs to be confirmed by other methods since confocal microscopy does not permit accurate quantification of the <30 nm distance.

## Conclusions

Although it remains to be fully characterized how ER-mitochondria contacts are maintained and regulated, it is becoming increasingly clear that MERC provide an important platform to intertwine various signaling pathways to carry out many cellular processes of importance to neuronal function including the regulation of calcium homeostasis, phospholipid synthesis and exchange, mitochondrial biogenesis and dynamics and apoptosis. It is interesting that disturbance in MERC are involved in most of the neurodegenerative diseases studied such as AD, PD and ALS as discussed above. Indeed, disturbance in MERC provides a connection for the various seemingly disparate features of these neurodegenerative diseases which may suggest that the disturbance in MERC may serve as a common convergent mechanism underlying neurodegeneration. Admittedly appealing, this hypothesis faces several challenges. First, the many conflicting observations, likely due to the different methods used, need to be reconciled. For example, the fundamental involvement of Mfn2 in the ER-mitochondria tethering is under hot debate and the effects of PS2 on MAM need clarification. Secondly, it appears that both upregulated and disrupted MAM functions are implicated in neurodegenerative diseases, even in the same disease. For example, VAPB-P56S mutant caused enhanced ER-mitochondria crosstalk while TDP43 and FUS disrupted ER-mitochondria contacts. While both upregulated and disrupted MAM function could lead to cellular dysfunction and neurodegeneration, this highlights the need to characterize the effects of specific neurodegenerative disease insults on the ER–mitochondria axis. Thirdly, the detailed mechanism of MAM disturbance in these conditions needs to be worked out. Is there a common mechanism? TDP43 and FUS disrupt the VAPB-PTPIP51 tethering through GSK3β activation. While GSK3β is activated in many of these neurodegenerative diseases, it may not elicit the same outcome since PS1 mutation caused GSK3β activation [79] but led to increased ER-mitochondria tethering [80]. Fundamentally, therefore, the pathophysiological relevance of these observations also need to be firmly established.

## Abbreviations

AD: Alzheimer’s disease
ALS: Amyotrophic lateral sclerosis
Aβ: Amyloid-β
Bap31: B-cell receptor-associated protein 31
CoA: Coenzyme A
DLP1: Dynamin-like protein
ER: Endoplasmic reticulum
FACL4: Long-chain-fatty-acid-CoA ligase 4
Fis1: Mitochondrial fission 1 protein
FTD: Frontotemporal dementia
FUS: Fused in sarcoma
grp75: Glucose-regulated protein 75
GSK3β: Glycogen synthase kinase 3β
IP3R: Inositol 1,4,5-trisphosphate receptor
MAMs: Mitochondrial associated membrane
MCU: Mitochondrial calcium uniporter
MERC: Mitochondria-ER contact/crosslink/crosstalk/communication
Mfn: Mitofusin
mPTP: Mitochondrial permeability transition pore
PACS-2: Phosphofurin acidic cluster sorting protein 2
PARK2: Parkin
PD: Parkinson’s disease
PS: Presenilin
PSS: Phosphatidylserine synthase
PTPIP51: Protein tyrosine phosphatase-interacting protein-51
SERCA: Sarco–endoplasmic reticulum Ca^2+^ ATPase
Sig-1R: Sigma1R
SOD1: Superoxide dismutase 1
TCA: Tricarboxylic acid cycle
TDP-43: TAR DNA-binding protein 43
VAPB: Vesicle-associated membrane protein-associated protein B
VDAC: Voltage-dependent anion channel
